# Allogeneic haematopoietic stem cell transplantation for refractory perforating intestinal Behçet disease in a patient with aplastic anaemia: A case report

**DOI:** 10.1097/MD.0000000000049997

**Published:** 2026-07-31

**Authors:** Xiuyun Rao, Xu Luo, Li Dai, Qian Wang

**Affiliations:** a Guizhou Provincial Key Laboratory for Digestive System Diseases, Guizhou Medical University and Affiliated Hospital of Guizhou Medical University, Guiyang, Guizhou, China; b Guizhou Provincial Key Laboratory for Digestive System Diseases, Affiliated Hospital of Guizhou Medical University, Guiyang, Guizhou, China.

**Keywords:** allogeneic hematopoietic stem cell transplantation, aplastic anemia, bone marrow failure, intestinal Behçet disease, intestinal perforation

## Abstract

**Rationale:**

Aplastic anemia (AA) rarely coexists with intestinal Behçet disease (iBD), and the post-partum period may further complicate its expression. Managing refractory iBD with recurrent perforation on a background of long-standing AA is challenging, and evidence on optimal treatment is limited.

**Patient concerns:**

A 42-year-old woman with non-severe aplastic anemia (NSAA) diagnosed in 2005 (AA duration ≈ 20 years) developed recurrent fever, oral ulcers, and penetrating ulcers of the ileocaecum and terminal ileum shortly after cesarean delivery in June 2019, with one episode of vulvar ulceration.

**Diagnoses:**

Histology showed transmural vasculitis with focal obliterative endarteritis, confirming iBD; HLA-B51 was negative. Pre-transplant marrow was markedly hypocellular without dysplasia, and karyotype (46,XX), flow cytometry, myelodysplastic syndrome/AML and myeloproliferative neoplasm gene panels, and paroxysmal nocturnal hemoglobinuria screening were all negative, supporting acquired AA rather than hypoplastic myelodysplastic syndrome.

**Interventions:**

Despite corticosteroids, tacrolimus, tofacitinib, and tocilizumab, she sustained 2 intestinal perforations (May and September, 2023). As iBD was refractory and AA had progressed to very severe AA, she underwent human leukocyte antigen (HLA)-matched sibling peripheral-blood allogeneic hematopoietic stem cell transplantation (allo-HSCT) in June 2024 with fludarabine/busulfan/cyclophosphamide/ anti-thymocyte globulin conditioning.

**Outcomes:**

Neutrophil and platelet engraftment occurred around D+11 and D+15, with 100% donor chimerism at D+16 and no acute or chronic graft-versus-host disease; only transient, self-limiting cytomegalovirus (CMV) and Epstein–Barr virus DNAemia occurred, without CMV disease or post-transplant lymphoproliferative disorder. Blood counts recovered progressively, with hemoglobin 127 grams/L and platelets 118 × 10^9^/L at the last follow-up (≈12 months), only mild residual cytopenia, transfusion independence, and sustained remission of iBD on maintenance immunosuppression. She then returned to work and was lost to follow-up.

**Lessons:**

This case suggests that allo-HSCT may be an effective option for selected patients with AA and refractory iBD, achieving sustained remission of both the marrow failure and the intestinal disease. As this is a single case with limited follow-up (≈12 months), longer follow-up and additional cases are needed.

## 1. Introduction

Aplastic anemia (AA) is an immune-mediated bone marrow failure syndrome in which autoreactive CD4^+^ and CD8^+^T cells drive Fas–FasL-mediated apoptosis of hematopoietic stem and progenitor cells, producing pancytopenia and markedly hypocellular marrow.^[1–3]^ Its annual incidence is only about 2 per million in Western populations, and although higher in East Asia, AA remains a rare haematological disease.^[4]^ Behçet disease (BD) is a chronic multisystem vasculitis characterized by recurrent oral ulcers (≥3 episodes per year) accompanied by genital ulcers, uveitis, or typical dermatologic findings.^[5]^ Intestinal Behçet disease (iBD) is the gastrointestinal subtype, characterized by deep, “punched-out,” transmural ulcers of the ileocaecum and terminal ileum.^[6–8]^ These ulcers are prone to perforation and frequently require surgery, with up to approximately 37% of patients undergoing emergency surgery for complications such as perforation.^[9]^

The coexistence of AA and iBD is extremely rare. In a retrospective analysis of 79 patients with BD, Ahn et al identified only 13 with bone marrow failure – of whom only 5 had AA – and intestinal involvement was significantly more frequent in the marrow-failure group (61.5% vs 13.6%, *P* = .001).^[10]^ Reported AA/iBD overlap has most often been associated with myelodysplastic syndrome (MDS) carrying trisomy 8, whereas non-clonal AA coexisting with iBD is even less frequently described.^[11–13]^ The 2 conditions share overlapping disturbances of T-cell homeostasis,^[14–17]^ which complicates clinical management.

For patients with coexisting AA and iBD, treatment usually combines the standard regimens for each disease: immunosuppression (corticosteroids, ciclosporin, anti-thymocyte globulin) and hematopoietic support (thrombopoietin-receptor agonists) for AA^[1,3]^; and, for iBD, 5-aminosalicylic acid, corticosteroids, and immunosuppressants stratified by severity,^[18–20]^ with TNF-α or interleukin-6 inhibitors added in refractory cases.^[21,22]^ However, simply combining these strategies in a patient with an immune–hematological overlap has important limitations: prolonged immunosuppression may further aggravate marrow failure and increase infection risk, and current biologics suppress pathogenic T-cell activity only transiently, making durable disease control difficult.

Allogeneic hematopoietic stem cell transplantation (allo-HSCT) is a curative-intent treatment for severe AA^[23]^ and has more recently been used for selected refractory autoimmune diseases.^[24,25]^ By eliminating recipient autoreactive T cells through conditioning and reconstituting hematopoiesis and immunity with donor-derived stem cells, allo-HSCT may, unlike immunosuppression alone, simultaneously address the marrow failure of AA and the immune-mediated intestinal injury of iBD.^[24,26]^

Here we report a 42-year-old woman who, on a background of long-standing non-severe aplastic anemia (NSAA), developed postpartum recurrent oral ulcers and penetrating ileocecal and terminal ileal ulcers. After failure of conventional immunosuppression and tocilizumab and 2 intestinal perforations, she achieved sustained dual remission of iBD and AA following human leukocyte antigen (HLA)-matched sibling allo-HSCT, providing a reference for the management of this rare and complex overlap.

## 2. Ethical approval

Ethical review and approval were waived for this study by the Institutional Review Board of the Affiliated Hospital of Guizhou Medical University because this is a single retrospective case report and does not involve any experimental intervention. Written informed consent was obtained from the patient for publication of the clinical details, clinical images, and pathological material presented in this report.

## 3. Case summary

**Figure 1.**
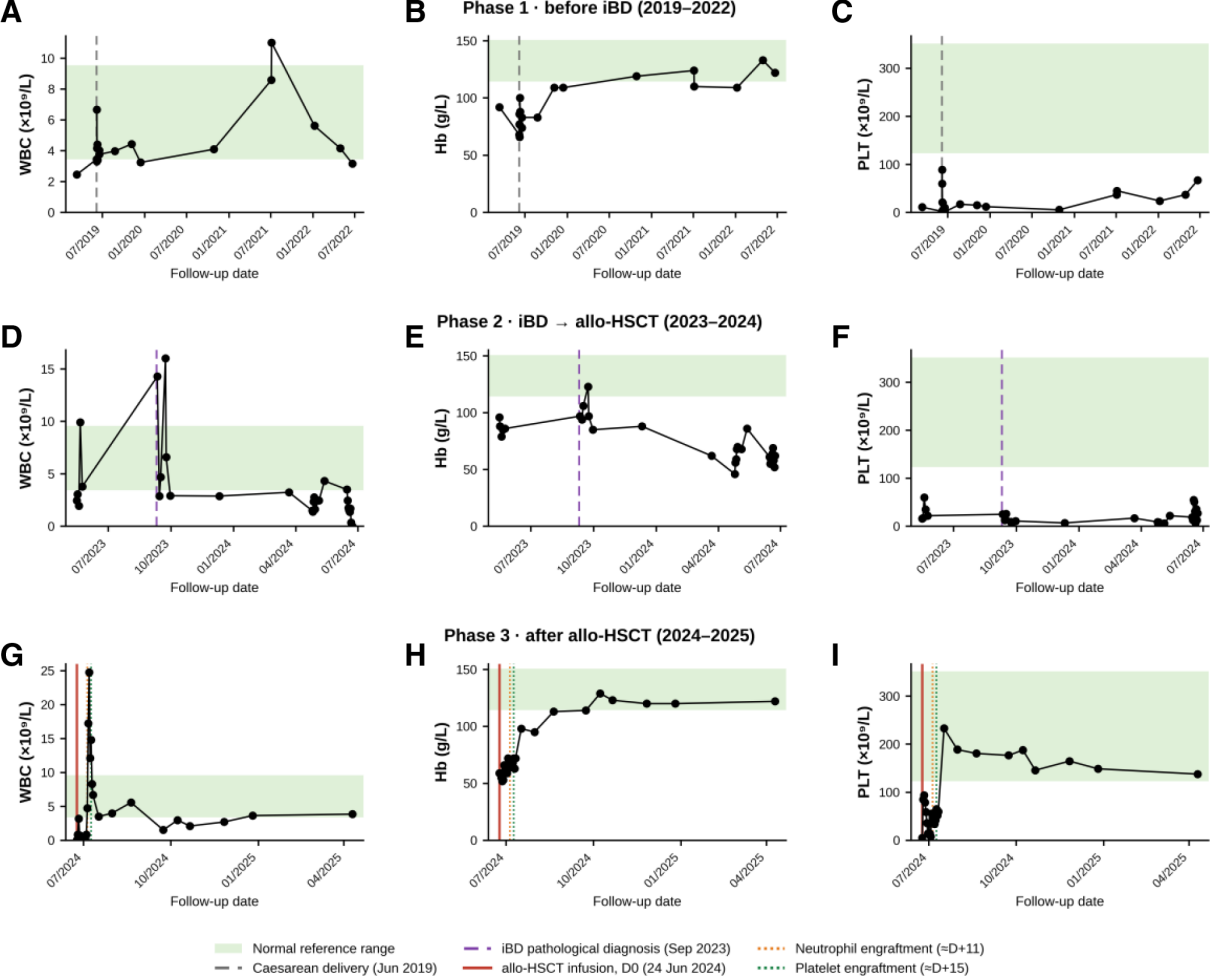
Dynamic changes in hematologic parameters across the disease course. The 9 panels are arranged in 3 rows × 3 columns; each row corresponds to one disease phase and, within each row, the 3 columns show (from left to right) the white blood cell count (WBC), hemoglobin (Hb), and platelet count (PLT). (A–C) Phase 1, before pathological confirmation of intestinal Behçet disease (iBD) (2019–2022); (D–F) Phase 2, from iBD diagnosis to allogeneic hematopoietic stem cell transplantation (allo-HSCT) (2023 to mid-2024); (G–I) Phase 3, after allo-HSCT (from June 2024). Accordingly, WBC is shown in (A), (D), and (G); Hb in (B), (E), and (H); and PLT in (C), (F), and (I). The x-axis indicates the follow-up date, and the shaded bands denote the normal reference ranges (WBC 3.5–9.5 × 10^9^/L; Hb 115–150 g/L; PLT 125–350 × 10^9^/L). Vertical lines mark key events: cesarean delivery (gray, June 2019), pathological diagnosis of iBD (purple, September 2023), allo-HSCT infusion (red, day 0 [D0], June 24,2024), neutrophil engraftment (orange, ≈D+11), and platelet engraftment (green, ≈D+15). allo-HSCT = allogeneic hematopoietic stem cell transplantation, Hb = hemoglobin, iBD = intestinal Behçet disease, PLT = platelet count, WBC = white blood cell count.

A 42-year-old woman first diagnosed with NSAA in 2005 (AA duration ≈ 20 years) developed intestinal Behçet disease (iBD) following cesarean delivery in June 2019. Her clinical course, treatments and outcomes are summarized in Table 1; dynamic haematological parameters are shown in Figure 1; the pre-transplant differential work-up and post-partum lymphocyte-subset data are provided in Supplementary Tables S1 and S2, Supplemental Digital Content 1.

**Table 1:** Clinical characteristics and treatment interventions.

Item	Detail
Sex/ Age	Female/ 42 yr
Diagnosis	Non-severe aplastic anemia (NSAA) with refractory perforating intestinal Behçet disease (iBD)
Duration of AA	≈20 yr (initial diagnosis 2005)
Duration of iBD	≈6 yr (from first post-partum oral ulcer, August 2019); pathological confirmation September 2023
Main clinical features	Recurrent oral ulcers (≥3/yr); a single vulvar ulcer; multiple penetrating ulcers of the ileocaecum and terminal ileum with 2 perforations; acute peritonitis; severe anemia and thrombocytopenia
Early post-partum immune phenotype (August 2019)	CD3^+^ 87.89% ↑, CD8^+^ 43.13% ↑, CD4^+^ 37.72%, NK 4.59% ↓, CD4/CD8 0.87 ↓
HLA typing	A*02:07/11:01; B*37:01/46:01 (B51 negative); C*01:02/06:02; DQB1*05:01/05:02; DRB1*10:01/14:54
MDS/MPN clonal screening	Karyotype 46,XX; blast region 0.27%; DNMT3A, JAK2-V617F, CALR, MPL, TP53, ASXL1, RUNX1, SF3B1, SRSF2, U2AF1, etc – all negative; PNH clone (CD59/FLAER) negative
Prior treatments	Immunosuppressants: corticosteroids, CsA, TAC, CTX, THD, LEN, TOF; hematopoietic support: androgens, danazol, TPO-RAs (eltrombopag, hetrombopag); biologic: TCZ; intestinal therapy: 5-ASA (mesalazine)
Allogeneic HSCT	June 24–25, 2024; HLA 10/10 matched-sibling (younger brother) donor; conditioning fludarabine/busulfan/cyclophosphamide/ATG; GVHD prophylaxis CsA + MMF; total CD34^+^ 6.41 × 10^6^/kg; adjuvant umbilical-cord-derived MSC, 6 units
Engraftment	Neutrophil ≈ D+11 (July 5, 2024); platelet ≈ D+15 (July 9, 2024); D+16 marrow smear confirmed reconstitution; X&Y-FISH 100% donor chimerism at D+16, with sustained trilineage engraftment thereafter
Peri-transplant complications	Febrile neutropenia (controlled with imipenem + vancomycin), oral ulcers, perianal lesions, hypoalbuminaemia; NO acute/chronic GVHD; transient self-limiting CMV/EBV DNAemia (no CMV disease or PTLD); NO VOD
Current maintenance therapy	CsA 100 mg b.i.d.; MMF 0.18 g b.i.d.; prednisone acetate 5 mg q.d.; hetrombopag 15 mg q.d.; maintenance immunosuppression was still ongoing at the last follow-up (June 25, 2025) (hetrombopag is a thrombopoietin-receptor agonist, not immunosuppression)
Follow-up duration	≈12 mo (until June 25, 2025); the patient subsequently returned to work and was lost to follow-up (further data pending)
Clinical response	iBD: sustained clinical remission, no recurrent perforation or ulceration. AA: durable donor engraftment (100% chimerism at D+16; sustained trilineage recovery), transfusion-independent; progressive haematological recovery (Hb 122 g/L, PLT 138 × 10^9^/L by Apr 2025; Hb 127 g/L, PLT 118 × 10^9^/L by June 2025), with mild residual cytopenia at the last follow-up

5-ASA = 5-aminosalicylic acid, AA = aplastic anemia, ATG = anti-thymocyte globulin, CMV = cytomegalovirus, CsA = ciclosporin, CTX = cyclophosphamide, EBV = Epstein–Barr virus, GVHD = graft-versus-host disease, HLA = human leukocyte antigen, iBD = intestinal Behçet disease, LEN = lenalidomide, MDS = myelodysplastic syndrome, MMF = mycophenolate mofetil, MPN = myeloproliferative neoplasm, MSC = mesenchymal stem cell, NK = natural killer, PNH = paroxysmal nocturnal hemoglobinuria, TAC = tacrolimus, TCZ = tocilizumab, THD = thalidomide, TOF = tofacitinib, TPO-RA = thrombopoietin-receptor agonist, VOD = veno-occlusive disease.

### 3.1. Medical history before delivery (2005–2019)

She presented around 2005 with fatigue and subcutaneous bleeding; bone marrow biopsy showed markedly decreased cellularity, confirming NSAA. She received supportive therapy, including corticosteroids, androgens, ciclosporin, cytokines, and intermittent transfusion. Regular danazol from 2016 produced a satisfactory hematological response and was later stopped in preparation for pregnancy. Before pregnancy, she had occasional ecchymoses and mildly prolonged bleeding after minor trauma.

### 3.2. Caesarean delivery and early postpartum course (2019)

On June 6, 2019 she underwent a lower-segment cesarean section, with peri-operative red-cell and platelet support. On post-partum day 12 (June 18, 2019), she developed acute airway obstruction due to a pharyngeal blood clot, which was removed bronchoscopically. Danazol (0.2 g 3 times daily) was resumed. These peri-partum events – transfusion dependence and mucosal bleeding causing airway obstruction – indicated severe thrombocytopenia and a fragile hematopoietic reserve related to her underlying AA.

About 2 months post-partum (August 2019), she first developed recurrent fever (up to 40°C), sore throat, and oral ulcers, with moderate anemia, severe thrombocytopenia (platelets 17 × 10^9^/L), and markedly raised ferritin (1761 ng/mL). At this time, lymphocyte-subset analysis showed a clear disturbance of T-cell homeostasis: CD3^+^ 87.89% (↑), CD8^+^ 43.13% (↑), CD4^+^ 37.72%, natural killer 4.59% (↓), and CD4/CD8 ratio 0.87 (↓) (Supplementary Table S2, Supplemental Digital Content 2). These changes persisted and progressed on repeat testing in December 2019 (CD4/CD8 0.65), showing a pattern of CD8^+^ T-cell predominance with reduced natural killer cells. Symptoms improved after anti-infective therapy, local treatment of mucosal lesions, and transfusion. Because of the recurrent oral ulcers, BD was considered, and methylprednisolone (40 mg/day) was added. Platelet counts subsequently declined further, reaching a nadir of 5 × 10^9^/L in late 2020, while white-cell and hemoglobin levels remained relatively stable with anti-infective and transfusion support (Fig. 1A–C).

### 3.3. Recurrent intestinal perforation and pathological confirmation of iBD (2022–2023)

**Figure 2.**
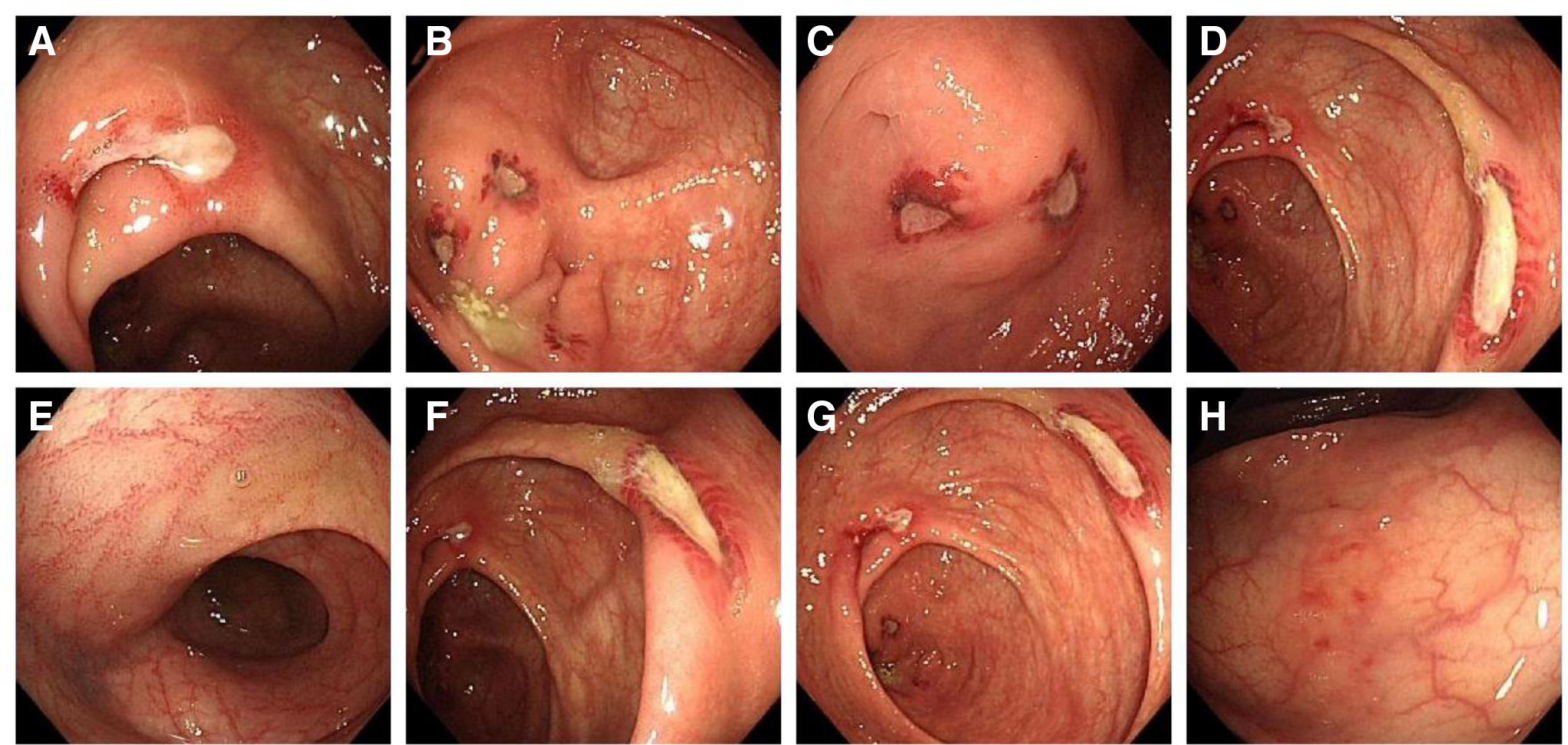
Colonoscopic findings (June 2022). (A) Ulcer of the ileocecal valve; (B) cecal ulcer; (C) ulcer at the appendiceal orifice and the surrounding area; (D) ascending colon ulcer; (E) terminal ileum; (F) ascending colon ulcer; (G) ulcers in the ascending colon and cecum; (H) ulcer in the ascending colon.

Colonoscopy in April 2022 revealed erosive inflammation of the ileocaecal region and multiple colonic ulcers. Follow-up 2 months later showed partial mucosal healing but persistent ulceration (Fig. 2); biopsy confirmed chronic intestinal inflammation. A concurrent autoimmune-liver and connective-tissue antibody screen was negative (Supplementary Table S1, Supplemental Digital Content 1).

**Figure 3.**
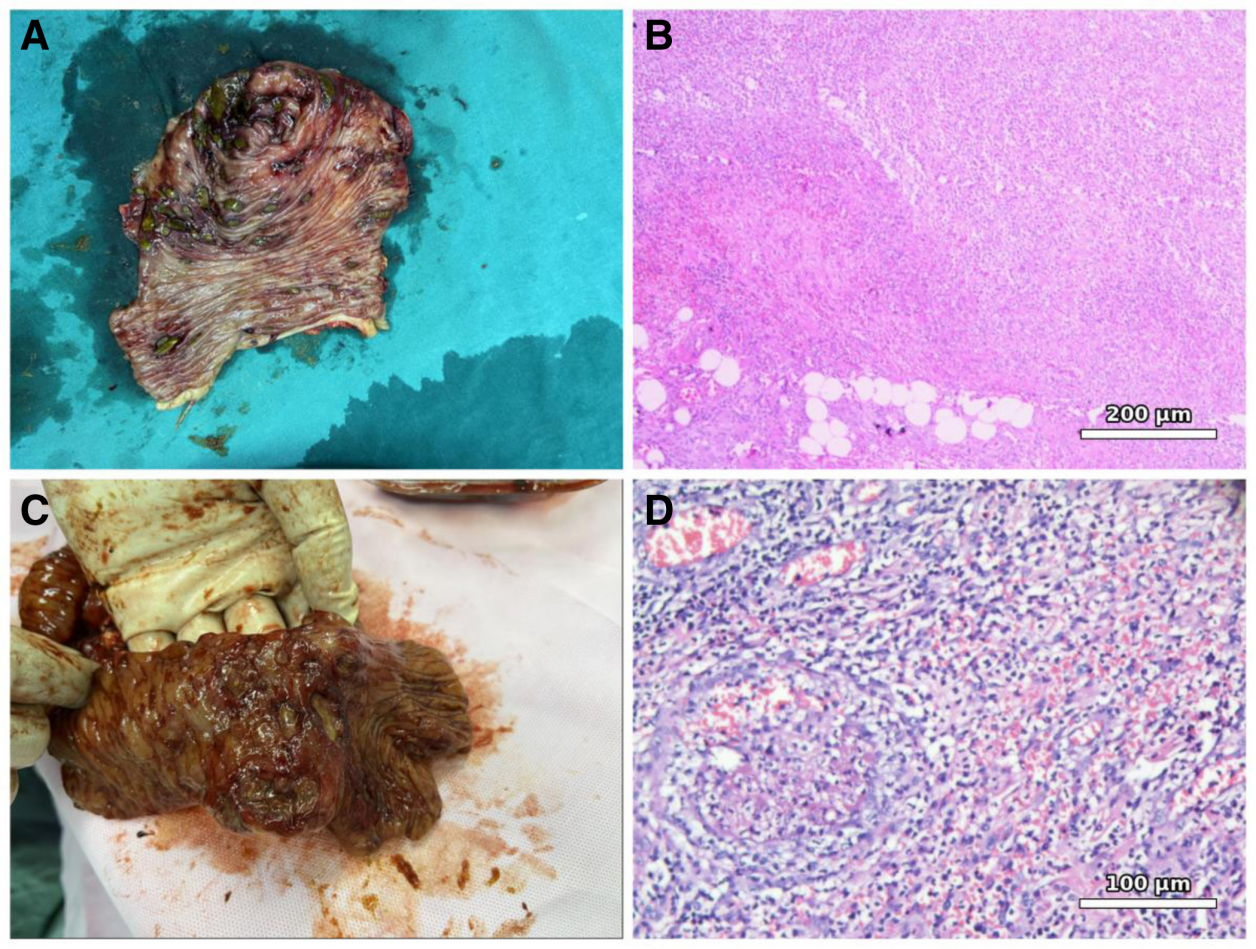
Intra-operative and histopathological findings of the 2 perforation episodes. (A) May 17, 2023: gross specimen from the ileocecal resection, showing multiple discrete, sharply demarcated (“punched-out”) ulcers. (B) Histopathology of the resected ileocaecal specimen: full-thickness chronic ulceration with granulation-tissue hyperplasia and chronic inflammation with erosion at the resection margins (hematoxylin and eosin; scale bar = 200 µm). (C) September 10, 2023: gross specimen from the resection of the ileum (50 cm proximal and distal to the anastomosis) and the transverse colon, with large penetrating ulcers. (D) Histopathology of the resected intestine: severe transmural chronic inflammation with giant ulceration, fibrinoid necrotizing vasculitis, and focal obliterative endarteritis, consistent with intestinal Behçet disease (hematoxylin and eosin; scale bar = 100 µm).

On May 17, 2023 she developed acute peritonitis from a perforated ileocaecal ulcer and underwent laparoscopic-converted ileocaecal resection; histology showed full-thickness ulceration with granulation-tissue hyperplasia (Fig. 3A and B). Rheumatology considered intestinal involvement by BD; HLA-B51 was negative. She was discharged on prednisone acetate and tofacitinib. During this period, she had a single episode of vulvar ulceration that healed spontaneously without specific treatment and did not recur. Taken together with the recurrent oral ulcers and typical intestinal involvement, the findings were consistent with iBD.

Alternative causes of penetrating ileocaecal ulceration were systematically considered. Intestinal tuberculosis was excluded by negative mycobacterial PCR on the May 2023 resection specimen and persistently negative T-SPOT.TB on follow-up. The histological combination of fibrinoid necrotizing transmural vasculitis with focal obliterative endarteritis, together with the absence of epithelioid granulomata or skip lesions, argued against Crohn disease as well as intestinal tuberculosis. Ischaemic and drug-related ulceration were excluded, as the patient had no history of long-term non-steroidal anti-inflammatory drug use and no ischemic vascular event, and the histological vasculitis was inconsistent with ischemic injury. Cytomegalovirus colitis was considered unlikely in the absence of corresponding clinical, endoscopic, or histological features. A comprehensive autoimmune antibody screen was negative (Supplementary Table S1, Supplemental Digital Content 1). Together with recurrent oral and vulvar ulceration, these findings satisfied the diagnostic criteria for iBD. Diagnosis was made according to the 2014 International Criteria for Behçet Disease^[5]^: the patient scored 4 points – recurrent oral aphthosis (2) and genital (vulvar) aphthosis (2) – meeting the threshold of ≥4, whereas ocular, cutaneous, neurological, and vascular manifestations were absent throughout the disease course, and pathergy testing was not performed. She therefore did not fulfill the more restrictive 1990 International Study Group criteria, which require recurrent oral ulceration plus at least 2 additional features, but did satisfy the more sensitive International Criteria for Behçet Disease criteria. The gastrointestinal phenotype – deep, “punched-out” ileocaecal and terminal-ileal ulcers with the pathological hallmark of fibrinoid necrotizing transmural vasculitis and focal obliterative endarteritis – was consistent with iBD as defined by the 2020 consensus recommendations.^[18]^

In September 2023 she again developed abdominal pain; endoscopy showed multiple ulcers at the anastomosis and ileum, suspicious for BD. She was admitted to rheumatology, where interleukin-6 was markedly elevated (140.6 pg/mL), and tocilizumab was started. On September 10, 2023 she developed a further perforated ulcer and underwent resection of the ileum (50 cm proximal and distal to the anastomosis) and transverse colon. Histology showed severe chronic transmural inflammation with giant ulceration, fibrinoid necrotizing transmural vasculitis, and focal obliterative endarteritis, confirming iBD (Fig. 3C and D). She was discharged on tacrolimus, prednisone acetate, mesalazine, lenalidomide, and hetrombopag.

### 3.4. Pre-transplant haematological reevaluation and differential diagnosis (March 2024)

Bone marrow aspiration and biopsy performed on March 22, 2024 demonstrated marked hypocellularity, with only scant residual hematopoietic tissue. Histological sections showed predominantly fatty marrow spaces with occasional granulocytic (MPO^+^), erythroid (CD235a^+^), and megakaryocytic (CD61^+^) elements, and rare CD34^+^ progenitor cells; reticulin staining showed grade 0 fibrosis. The smear showed markedly decreased cellularity (myeloid:erythroid ratio 7.8), no megakaryocytes, and only sporadic platelets, with no blasts or abnormal infiltrate.

Karyotyping showed a normal female karyotype (46,XX [2]; few analyzable metaphases). Flow cytometry showed a blast region of 0.27% with no aberrant myeloid/lymphoid population and no evidence of acute leukaemia or an MDS-associated phenotype. An MDS/AML gene panel (DNMT3A-R882, TP53 [exons 4–9], SF3B1, SRSF2, U2AF1, ASXL1, RUNX1, JAK2-V617F, JAK2 exon 12, CALR exon 9, MPL-W515, FLT3-ITD/D835, NPM1, CEBPA, EZH2-Y646, IDH1/2) was negative, and an myeloproliferative neoplasm panel (eight CALR exon-9 variants and JAK2-V617F) was negative. Paroxysmal nocturnal hemoglobinuria (PNH)-clone screening (CD59/ fluorescein-labeled aerolysin variant) was negative. A full antinuclear antibodypanel, an anti-neutrophil cytoplasmic antibody panel (including anti-glomerular basement membrane antibody), anti-cardiolipin antibodies, and autoimmune-liver antibodies were all negative (antinuclear antibody and anti-neutrophil cytoplasmic antibody repeated in May 2024), and chest CT, abdominal MRI, echocardiography, electrolytes, and liver/renal function were unremarkable (Supplementary Table S1, Supplemental Digital Content 1).

Taken together, the markedly hypocellular marrow, absence of dysplastic morphology or excess blasts, lack of clonal cytogenetic or molecular abnormalities, negative PNH clone, comprehensively negative autoimmune screen, and negative flow cytometry were most consistent with acquired AA, with no morphological, cytogenetic, or molecular evidence of MDS at evaluation.

### 3.5. Decision for transplantation and peri-transplant course (May–July, 2024)

On May 13, 2024 she was re-admitted with severe anemia (Hb 46 g/L) and very severe thrombocytopenia (platelets 4 × 10^9^/L), meeting criteria for very severe AA (Fig. 1D–F). HLA typing was A*02:07/11:01, B*37:01/46:01, C*01:02/06:02, DQB1*05:01/05:02, DRB1*10:01/14:54; her younger brother was a 10/10 matched related donor. After multidisciplinary discussion, allo-HSCT was recommended as the curative-intent option, using peripheral-blood stem cells with fludarabine/busulfan/cyclophosphamide/ anti-thymocyte globulin (ATG) conditioning and veno-occlusive disease (VOD) prophylaxis. Conditioning began on June 14, 2024 with hydration, alkalinisation, hepatoprotection, prophylactic imipenem, vancomycin, and voriconazole, and alprostadil for veno-occlusive disease/ sinusoidal obstruction syndrome prophylaxis.

On June 24–25, 2024 she received peripheral-blood stem cells from her HLA-matched brother (CD34^+^ 3.27 and 3.14 × 10^6^/kg; total ≈ 6.41 × 10^6^/kg). Graft-versus-host disease (GVHD) prophylaxis was ciclosporin plus mycophenolate mofetil, and 6 units of umbilical-cord-derived mesenchymal stem cells were infused as an adjunct on 28 June. Early post-transplant, she developed pancytopenia, febrile neutropenia (peak 39.8°C, controlled after escalation to imipenem plus vancomycin), oral ulcers, perianal lesions, and hypoalbuminaemia, managed with irradiated blood products, recombinant bovine basic fibroblast growth factor mouthwash, albumin, and nutritional support.

The absolute neutrophil count fell to a nadir of 0.02–0.06 × 10^9^/L around D+6 to D+9, then recovered: by approximately D+11 (July 5, 2024), white cells and neutrophils rose together (ANC ≥ 0.5 × 10^9^/L for 3 consecutive days), meeting neutrophil-engraftment criteria, and platelets became transfusion-independent and remained ≥ 20 × 10^9^/L for 7 consecutive days by approximately D+15 (July 9, 2024) (Fig. 1G–I). A D+16 marrow smear showed early hematopoietic reconstitution consistent with peripheral recovery.

No acute or chronic GVHD, VOD, or BK viruria occurred. Peripheral-blood cytomegalovirus (CMV) and Epstein–Barr virus (EBV) DNA were monitored serially after transplantation; low-level DNAemia was detected intermittently – EBV first on D+25 (July 19, 2024) and CMV on D+38 (August 1, 2024), each clearing on subsequent testing, with further transient positivity during the first post-transplant year while the patient remained on immunosuppression. These episodes were self-limiting and did not require preemptive antiviral therapy, and there was no progression to CMV end-organ disease or EBV-associated post-transplant lymphoproliferative disorder through the last assessment (June 25, 2025). Donor chimerism by X&Y-FISH was 100% (1000/1000 interphase nuclei) at D+16; thereafter, sustained trilineage recovery and transfusion independence indicated maintained donor-derived hematopoiesis, although chimerism was not re-assessed before the patient was lost to follow-up.

On discharge she continued maintenance therapy with ciclosporin 100 mg twice daily, mycophenolate sodium 0.18 g twice daily, prednisone acetate 5 mg daily, and hetrombopag 15 mg daily, with regular monitoring of blood counts, liver/renal function, and chimerism.

### 3.6. Follow-up and outcome

**Figure 4.**
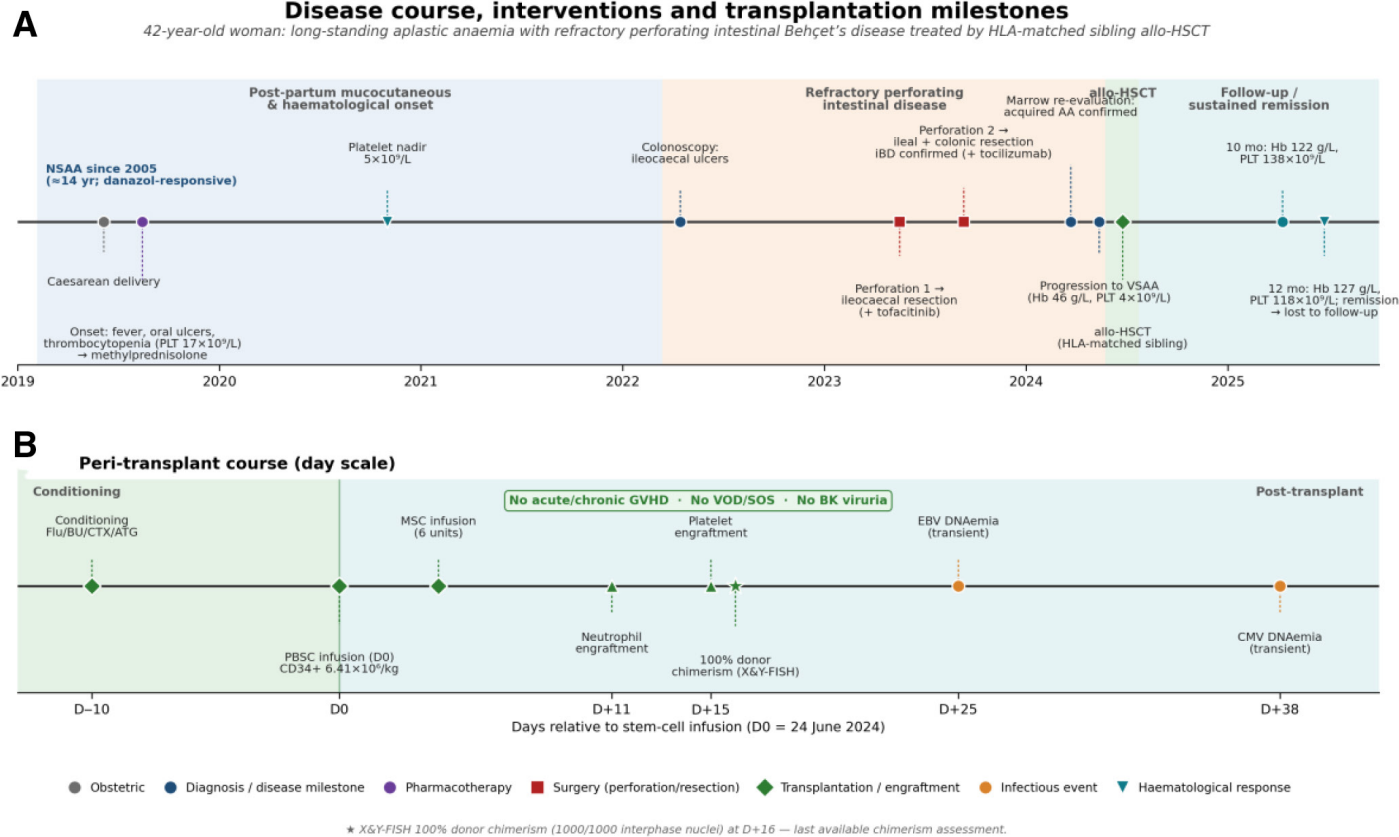
Disease course, interventions, and transplantation milestones. (A) Overview timeline (2019–2025): post-partum onset of mucocutaneous and haematological disease; refractory perforating intestinal Behçet disease with 2 intestinal perforations requiring resection; progression of aplastic anemia to very severe aplastic anemia; HLA-matched sibling allo-HSCT; and follow-up (on a background of non-severe aplastic anemia present since 2005, ≈14 yr before the onset of intestinal Behçet disease in 2019). (B) Peri-transplant course on a day scale (D0 = stem-cell infusion, June 24, 2024): conditioning (fludarabine/busulfan/cyclophosphamide/antithymocyte globulin), peripheral-blood stem-cell infusion (CD34^+^ 6.41 × 10^6^/kg), adjunctive umbilical-cord-derived mesenchymal stem-cell infusion, neutrophil (≈D+11) and platelet (≈D+15) engraftment, 100% donor chimerism by X&Y-fluorescence in situ hybridization (FISH) at D+16, and transient, self-limiting Epstein–Barr virus (D+25) and cytomegalovirus (D+38) DNAemia; there was no acute or chronic graft-versus-host disease, no veno-occlusive disease/sinusoidal obstruction syndrome, and no BK viruria. At 10 and 12 mo, the patient was transfusion-independent (hemoglobin 122 g/L, platelets 138 × 10^9^/L, then hemoglobin 127 g/L, platelets 118 × 10^9^/L) with sustained remission of both diseases, after which she returned to work and was lost to follow-up. HLA = human leukocyte antigen, HSCT = hematopoietic stem cell transplantation.

She achieved sustained hematopoietic engraftment with subsequent recovery of peripheral-blood counts. No further gastrointestinal perforation, ulcer recurrence, or systemic inflammatory flare occurred, and oral and vulvar ulcers did not recur. By approximately 10 months (April 10,2025), white cells were 3.86 × 10^9^/L, hemoglobin 122 g/L, and platelets 138 × 10^9^/L (Fig. 1G–I); abdominal pain had resolved and functional status had returned to baseline, with transfusion independence. At the most recent follow-up (June 25,2025, ≈12 months post-transplant), hemoglobin had risen further to 127 g/L, with white cells 4.92 × 10^9^/L and platelets 118 × 10^9^/L; there was mild residual neutropenia (neutrophils 1.62 × 10^9^/L) and borderline thrombocytopenia, together with a relative lymphocytosis (lymphocytes 53.7%) and monocytosis (monocytes 12.4%), a pattern commonly seen during post-transplant immune reconstitution. She remained on maintenance immunosuppression (ciclosporin, mycophenolate sodium, and prednisone acetate, together with hetrombopag) and transfusion-independent, with iBD in sustained clinical remission and AA showing maintained donor-derived hematopoiesis. She subsequently returned to work and resumed normal daily activities, after which she was lost to follow-up; further follow-up data – including peripheral-blood counts and donor chimerism after any tapering of immunosuppression – will be supplemented when the patient can be reassessed. The overall disease course, surgical interventions, and post-transplant haematological and clinical response are summarized in the timeline in Figure 4.

## 4. Discussion

We describe a patient with long-standing AA who developed refractory perforating iBD and achieved sustained remission after HLA-matched sibling allo-HSCT. Her AA had been present for nearly 20 years, presenting initially as chronic marrow failure; oral and intestinal lesions emerged postpartum and progressed to recurrent penetrating ileocaecal and terminal-ileal ulcers with 2 perforations.

### 4.1. Distinguishing acquired aplastic anemia from clonal Behçet-like myeloid disorders

Behçet-like intestinal disease associated with clonal myeloid disorders first had to be distinguished from coexisting acquired AA. MDS – particularly with trisomy 8 – can produce Behçet-like intestinal lesions.^[11–13]^ We therefore performed a systematic pre-transplant hematological evaluation: the marrow was markedly hypocellular without definite dysplasia, and karyotype, flow cytometry, an MDS/AML gene panel, and PNH screening were all normal, with no laboratory evidence of systemic autoimmune disease. These findings favor acquired AA coexisting with iBD rather than Behçet-like intestinal disease secondary to a clonal hematological disorder.

### 4.2. Atypical immunogenetic background

The immunogenetic background was also atypical: HLA-B51 was negative, in contrast to classic East-Asian BD, and the patient carried B*46:01 and DRB1*14. HLA allele distributions and their associations with hematological and autoimmune disease in Han Chinese have been reported,^[27–29]^ but whether these alleles are causally related to the AA/iBD phenotype here remains uncertain.

### 4.3. Comparison with previously reported cases

Compared with previously reported cases of Behçet-like intestinal disease associated with hematological disease, our patient differs in several important respects (Table 2). Most reported cases arise on a background of trisomy-8 myelodysplastic syndrome/neoplasm, tend to occur in older patients, and are usually HLA-B51-negative, and allo-HSCT (or azacitidine-based approaches followed by transplantation) has achieved remission of both the hematological and the intestinal disease.^[11,12,26,30]^ In contrast, our patient had non-clonal acquired AA with a normal karyotype and no trisomy 8, was HLA-B51-negative, and presented with perforating disease; nonetheless, HLA-matched sibling allo-HSCT achieved sustained remission of both conditions, extending the reported experience of allo-HSCT beyond clonal myeloid disorders.

**Table 2:** Comparison with reported cases of Behçet-like intestinal disease and haematological disease treated with, or evaluated for, allogeneic HSCT.

Study (yr)	Age/ Sex	Haematological disorder (cytogenetics)	Intestinal involvement (HLA-B51)	Treatment/ transplant	Outcome (follow-up)
Asano 2019^[12]^	18/ F	MDS with multilineage dysplasia (trisomy 8, 87%)	Caecal ulcer with microperforation (NR)	Ileocaecal resection; allogeneic PBSCT, mother donor	Complete resolution of BD manifestations; no severe adverse events
Ishii 2021^[26]^	45/ F	MDS (trisomy 8, sporadic by FISH)	Multiple colorectal ulcers (NR)	Azacitidine, then allo-HSCT (PBSC + bone marrow); massive lower-GI bleed early post-transplant	Long-term remission of MDS and Behçet-like disease
Wesner 2019^[11]^ (series, n = 11)	Median 75; M/F 0.8	Trisomy-8 MDS/MPN	GI involvement in 60% (NR)	Mainly immunosuppression/biologics; HSCT in a minority	Median survival 47 mo; immunosuppression frequently insufficient
Ahn 2008^[10]^ (cohort, n = 79 BD)	Korean BD cohort	Bone-marrow failure in 13 (AA in 5)	Intestinal involvement 61.5% in marrow-failure vs 13.6% (NR)	Varied; not transplant-focused	Intestinal ulceration and trisomy 8 associated with marrow failure
Present case	42/ F	Non-clonal acquired AA (NSAA → VSAA); normal karyotype 46,XX, no trisomy 8	Penetrating ileocaecal and terminal-ileal ulcers, 2 perforations (HLA-B51 negative)	Multiple immunosuppressants + biologics + two resections; HLA-matched sibling allo-PBSCT (fludarabine/busulfan/cyclophosphamide/ATG)	Sustained dual remission; transfusion-independent through ≈ 12 mo; 100% donor chimerism at D+16; no GVHD (lost to follow-up after ≈ 12 mo)

Most reported cases of Behçet-like intestinal disease arise in trisomy-8 MDS/MPN; the present case is distinguished by non-clonal acquired AA with a normal karyotype, HLA-B51 negativity and perforating disease treated by HLA-matched sibling allo-HSCT.

AA = aplastic anemia, ATG = anti-thymocyte globulin, BD = Behçet disease, FISH = fluorescence in situ hybridization, GI = gastrointestinal, GVHD = graft-versus-host disease, HSCT = hematopoietic stem cell transplantation, MDS = myelodysplastic syndrome, MPN = myeloproliferative neoplasm, NR = not reported, NSAA = non-severe AA, PBSCT = peripheral-blood stem cell transplantation, VSAA = very severe AA.

### 4.4. Post-partum onset and immune dysregulation

A further notable feature was disease onset in the early post-partum period. The patient had no BD-related manifestations during many years of AA, and her first oral ulcer occurred about 2 months post-partum, accompanied by an increased CD8^+^ fraction and a fallen CD4/CD8 ratio. Pregnancy and the post-partum period are recognized to be associated with shifts in immune status.^[31–34]^ The temporal sequence here suggests a possible association between post-partum immune change and the onset of iBD; however, because AA itself can produce similar T-cell-subset changes, the precise relationship remains difficult to establish.

### 4.5. Treatment rationale and transplantation outcome

The intestinal disease progressed despite corticosteroids, tacrolimus, tofacitinib, and tocilizumab, with 2 perforations, indicating that conventional immunosuppression and biologics failed to control disease activity; because progressive marrow failure had reached very severe AA, allo-HSCT was undertaken. Allo-HSCT is an established treatment for severe AA and is also used for selected refractory autoimmune diseases.^[23–25]^ With fludarabine/busulfan/cyclophosphamide/ATG conditioning, neutrophil and platelet engraftment occurred at approximately D+11 and D+15, and X&Y-FISH confirmed complete donor chimerism at D+16, with sustained trilineage recovery and transfusion independence thereafter; chimerism was not re-assessed before the patient was lost to follow-up. No acute or chronic GVHD occurred, and there was no further perforation or significant recurrence of intestinal symptoms, suggesting that allo-HSCT simultaneously improved the marrow failure and the intestinal disease in this patient.

Cord-derived mesenchymal stem cells were given peri-transplant; although mesenchymal stem cells may have immunomodulatory effects,^[35]^ their specific contribution to the outcome cannot be assessed in this single case.

### 4.6. Limitations

This report has limitations. It is a single-center, single-patient observation with limited follow-up (approximately 12 months of clinical and laboratory follow-up, after which the patient returned to work and was lost to follow-up), which constrains the strength and generalisability of the conclusions; in particular, donor chimerism was last documented at D+16, and the long-term durability of haematological recovery and the course after any discontinuation of immunosuppression remain to be confirmed. Updated follow-up will be reported when the patient can be reassessed. Tissue immunological and cytokine studies (including STAT3 activation) were not performed, so the understanding of the underlying mechanism remains based mainly on clinical observation. Nevertheless, the case suggests that, for selected patients with refractory iBD complicated by marrow failure, allo-HSCT may warrant further study as a treatment option.

## 5. Conclusion

We report a patient with long-standing AA who developed refractory perforating iBD and, after failure of multiple immunosuppressive and biologic therapies and recurrent perforation, achieved sustained remission following HLA-matched sibling allo-HSCT. Haematopoiesis recovered, intestinal symptoms did not recur, and no severe transplant-related complications occurred. This case suggests that allo-HSCT may be a feasible treatment option for selected patients with refractory iBD complicated by marrow failure. As a single case report, longer follow-up and additional cases are needed to evaluate efficacy and safety.

## Author contributions

**Conceptualization:** Xiuyun Rao.

**Data curation:** Xiuyun Rao, Xu Luo.

**Project administration:** Li Dai.

**Resources:** Xiuyun Rao, Xu Luo.

**Software:** Xiuyun Rao.

**Writing – original draft:** Xiuyun Rao.

**Writing – review & editing:** Li Dai, Qian Wang, Xu Luo.




