# Supplementary material for: Allogeneic haematopoietic stem cell transplantation for refractory perforating intestinal Behçet disease in a patient with aplastic anaemia: A case report
Source: Medicine (Baltimore). 2026 Jul 31;105(31):e49997. doi: 10.1097/MD.0000000000049997 (PMC13433067; doi:10.1097/MD.0000000000049997)
Supplement: Supplementary file 1 [file medi-105-e49997-s001.docx]

**Supplementary Table S1 Pre-transplantation differential diagnostic work-up.**

| **Investigation (date)** | **Result** | **Clinical implication** |
| --- | --- | --- |
| Karyotype (22 Mar 2024) | 46,XX [2] | Normal female karyotype; no MDS-defining lesion |
| Flow cytometry (22 Mar 2024) | Blast region 0.27%; no aberrant phenotype | Excludes acute leukaemia and MDS-associated populations |
| MDS/AML gene panel (22 Mar 2024) | DNMT3A-R882, TP53 (exons 4–9), SF3B1, SRSF2, U2AF1, ASXL1, RUNX1, JAK2-V617F, CALR exon 9, MPL-W515, FLT3-ITD/D835, NPM1, CEBPA, EZH2-Y646, IDH1/2 — all negative | Excludes MDS/AML clonal disorders |
| MPN molecular panel (22 Mar 2024) | Eight CALR exon-9 variants negative; JAK2-V617F negative | Excludes myeloproliferative neoplasms |
| PNH clone | CD59/FLAER negative | Excludes PNH / PNH-AA syndrome |
| ANA panel (Jan 2020 & May 2024) | ANA (1:100–1:10000), dsDNA, Sm, SSA, SSB, Ro-52, Scl-70, Jo-1, CENP-B, PCNA — all negative | Excludes SLE and connective-tissue disease |
| ANCA panel (Jan 2020 & May 2024) | pANCA, cANCA, MPO-ANCA, PR3-ANCA, anti-GBM — all negative (2024 by IIF and immunoblotting) | Excludes ANCA-associated vasculitis and anti-GBM disease |
| Anti-cardiolipin antibodies (Jan 2020) | ACA-IgM, IgG, IgA — all negative | Excludes antiphospholipid syndrome |
| Autoimmune-liver antibodies (Apr 2022) | SMA, anti-LKM, anti-LC-1, anti-SLA/LP, AMA, AMA-M2 — all negative | Excludes autoimmune hepatobiliary disease |
| Mycobacterial testing | Tissue mycobacterial PCR negative; T-SPOT.TB negative | Excludes intestinal tuberculosis |
| HLA-B51 | Negative | Distinct from classic East-Asian BD background |
| Full HLA typing (13 May 2024) | A*02:07/11:01; B*37:01/46:01; C*01:02/06:02; DQB1*05:01/05:02; DRB1*10:01/14:54 | 10/10 match with younger brother; B*46:01 and DRB1*14 present |
| Imaging | Chest CT, abdominal MRI, echocardiography, ultrasound — unremarkable | Excludes overt structural lesions |

*MDS = myelodysplastic syndrome; AML = acute myeloid leukaemia; PNH = paroxysmal nocturnal haemoglobinuria; FLAER = fluorescein-labelled aerolysin variant; ANA = antinuclear antibody; ANCA = anti-neutrophil cytoplasmic antibody; SLE = systemic lupus erythematosus; anti-GBM = anti-glomerular basement membrane antibody; IIF = indirect immunofluorescence; HLA = human leukocyte antigen; BD = Behçet’s disease; T-SPOT.TB = tuberculosis-specific interferon-γ release assay.*
