# Supplementary material for: Allogeneic haematopoietic stem cell transplantation for refractory perforating intestinal Behçet disease in a patient with aplastic anaemia: A case report
Source: Medicine (Baltimore). 2026 Jul 31;105(31):e49997. doi: 10.1097/MD.0000000000049997 (PMC13433067; doi:10.1097/MD.0000000000049997)
Supplement: Supplementary file 2 [file medi-105-e49997-s002.docx]

**Supplementary Table S2 Dynamics of lymphocyte subsets at two post-partum time points.**

| **Parameter** | **26 Aug 2019** | **20 Dec 2019** | **Reference range** |
| --- | --- | --- | --- |
| CD3+ (%) | 87.89 ↑ | 84.9 ↑ | 64.62–77.08 / 50–84 |
| CD3+CD8+ (%) | 43.13 ↑ | 48.9 ↑ | 24.81–35.99 / 15–44 |
| CD3+CD4+ (%) | 37.72 | 32.0 | 32.69–44.23 / 27–51 |
| CD16+CD56+ NK (%) | 4.59 ↓ | 4.7 | 6.38–12.46 / 0.5–30 |
| CD19+ B cells (%) | 7.35 | 8.7 | — |
| CD4/CD8 ratio | 0.87 ↓ | 0.65 ↓ | 1.04–1.72 / 0.71–2.78 |

*Lymphocyte-subset analyses were performed by flow cytometry on peripheral blood. The two reference ranges correspond to the two reporting laboratories used in Aug 2019 and Dec 2019. ↑ above reference; ↓ below reference. The progressive CD4/CD8 inversion (0.87 → 0.65), CD8+ expansion and NK reduction indicate a persistent disturbance of T-cell homeostasis emerging within two months post-partum.*
